# AI literacy and college students' innovative thinking: chain mediating roles of AI interaction perception and AI attitudes

**DOI:** 10.3389/fpsyg.2026.1838803

**Published:** 2026-07-10

**Authors:** Xiangui Bu, Wenqi Wang, Xuesong Ji, Yanjun Huang, Daoye Lu

**Affiliations:** 1 School of Competitive Sports, Shandong Sport University, Rizhao, China; 2 Graduate School of Shandong Sport University, Shandong Sport University, Jinan, China; 3 The School of Humanities, Arts and Education, Shandong Xiehe University, Jinan, China; 4 Department of Arts and Physical Education, Huanghe Science and Technology University, Zhengzhou, China

**Keywords:** AI attitudes, AI interaction perception, AI literacy, college student, innovative thinking

## Abstract

**Objective:**

Apart from progress in productivity, the rise of artificial intelligence (AI) technologies has increased requirements for talent cultivation in colleges and universities. Despite the rapid integration of artificial intelligence in higher education, the mechanisms through which AI literacy influences students' innovative thinking remain insufficiently understood. Based on the social cognitive and technology affordance theories, this study discusses the promoting effect of AI literacy on college students' innovative thinking and conducts an in-depth exploration of specific mechanisms of AI literacy that influence innovative thinking.

**Methods:**

This study employed a stratified random sampling approach, recruiting students from four universities. A total of 1,000 university students were invited to complete an online questionnaire to ensure adequate statistical power. The study adopted four standardized scales, namely, Artificial Intelligence Literacy Scale, Innovative Thinking Scale, Perceived Interactivity of Learner-AI Interaction Scale, and Artificial Intelligence Attitude Scale. SPSS 26.0 was used for statistical analysis, PROCESS macro was employed to test the proposed chain mediation model, and the bootstrap method was used to verify the significance of different paths.

**Results:**

The results revealed significant gender differences in AI attitudes and significant grade-level differences in perceived AI interaction. Significant correlations were found between AI literacy and innovative thinking, perceived AI interaction, and AI attitudes (*r* = 0.139, *p* < 0.001; *r* = 0.366, *p* < 0.001; *r* = 0.436, *p* < 0.001). AI interaction perception and AI attitudes individually played mediating roles between AI literacy and innovative thinking, respectively. Meanwhile, AI interaction perception and AI attitudes played chain mediating roles between AI literacy and innovative thinking.

**Conclusion:**

AI literacy can significantly positively predict the innovative thinking. It influences innovative thinking not only through the independent mediating roles of perceived AI interaction and AI attitudes but also through the sequential mediating pathway of AI literacy → AI interaction perception → AI attitudes → innovative thinking. The direct path is fully mediated once perceived AI interaction and AI attitudes are entered. These findings suggest that improving AI literacy is an effective strategy for enhancing university students' innovative thinking, and strengthening perceived AI interactions and attitudes may further amplify this effect.

## 1 Introduction

With the advent of the digital-intelligent era, the application scenarios of artificial intelligence (AI) in colleges and universities have become increasingly complex and diverse (Sui et al., 2025). AI has attracted widespread scholarly attention for its ability to automatically generate text, images, audios, and other contents, thus providing efficient application experiences for teachers and students. At the end of 2022, the emergence of ChatGPT rapidly drew global attention to generative AI (Fan and Liang, 2024). AI can provide personalized and efficient learning tutoring for college students and propose intelligent problem-solving ideas for university teachers (Zhao, 2024). Despite AI's significant application potential to higher education, various researchers have expressed concerns regarding privacy issues, security risks, technical feasibility, and acceptability in the application process, including potential conflicts with teaching quality in higher education (Shahzad et al., 2025). However, given AI's rapid development, numerous institutions of higher education have gradually realized that the tide of the AI era is irresistible and have actively explored ways to better integrate AI into existing education systems (Lim et al., 2025). Higher education institutions have gradually provided teachers and students with access to generative AI, and many universities have issued corresponding usage guidelines. An increasing number of teachers and students have accessed generative AI and applied it to teaching and research activities (Sun, 2025).

The widespread application of AI technology has not only improved the productivity of college teaching but also increased requirements for talent cultivation in higher education (Xu, 2024). Against the background that AI technology promotes the transformation and development of literacy among students, a need emerges for colleges and universities to pay more attention to the cultivation of high-order thinking such as innovation ability among students and cultivate a group of innovative talents adapted to the development of the digital-intelligent era (Chen and Li, 2026). As a core competence that can instigate changes, scholars have highlighted the importance of innovative thinking unprecedentedly (Zhang et al., 2025). Although scientific curriculum education and creative scientific training in higher education can stimulate students' innovative thinking, research on its continuous cultivation remains lacking.

On the one hand, given the gradual penetration of AI into all facets of life, the demand for talents in the labor market has undergone groundbreaking changes, and innovative thinking has become the core driving force of future competencies (Liang and Deng, 2025). The widespread application of intelligent technology has placed many traditional labor positions under pressure of displacement. College students with strong innovative thinking skills are better equipped to recognize the advantages and limitations of AI and utilize AI to amplify their creative work; in this manner, they explore new career positions and development opportunities within human machine collaboration environments (Zhao et al., 2025). On the other hand, the application of AI technology to smart medical care, intelligent transportation, intelligent manufacturing, and other fields has generated numerous new business models and employment opportunities (Zhang et al., 2025). The core competitiveness of these emerging fields lies in continuous technological innovation, which is inseparable from innovative talents (Lan et al., 2025). By cultivating innovative thinking, college students can better comprehend the development trend of these emerging technologies, discover opportunities, and thus become an important force that promotes industrial upgrading and economic development (He et al., 2026). Therefore, innovative thinking promotes the career development of college students as well as lays a solid spiritual foundation for social progress and scientific and technological development (Li et al., 2026).

A number of studies pointed out that AI literacy is an important carrier of the coupling and correlation between current AI technology and socioeconomic development (Ramírez-Correa et al., 2026). Therefore, college students should possess high levels of AI literacy in the processes of adapting to the technological development of the AI era and collaborating with AI (Deshen et al., 2026). Several studies attribute AI literacy to a collection of qualities such as knowledge and AI attitudes; usage skills; extended application; security; and ethics. AI literacy can promote high-quality employment among college students by improving career planning to align with the supply-and-demand structure of the labor force, enhancing job-seeking capabilities to optimize human resource allocation, elevating human capital to enhance the quality of labor supply, strengthening social capital level to optimize career development paths, and enhancing risk management skills to stimulate talent innovation potential (Jing and Liu, 2025). AI literacy can enhance or weaken students' ability to apply innovative thinking to professional tasks, thus influencing their preparation for future roles (Chisale, 2026). AI literacy enhances intrinsic motivation by meeting the psychological requirements of competence, autonomy, and relatedness. College students with high levels of AI literacy are likely to experience greater competence and confidence, thus promoting career development (Zehui et al., 2025).

Current surveys demonstrate the widespread use of AI in the daily lives of college students, typically using AI to improve learning progress and learning ability, which consequently enhances innovative thinking (Jing and Liu, 2025). A number of studies pointed out that a significant positive correlation exists between college students' AI literacy and innovative thinking, and the greater use of AI technology in daily life improves creativity in problem solving (Wang and Zhang, 2025). The reason underlying this result may be that AI literacy provide college students with easy access to emerging industries and technologies and cultivate their acceptance of new things, thus leading to high levels of innovative thinking (Yordudom et al., 2026). Two explanations are plausible. First, AI literacy promotes the development of critical thinking among college students when processing diverse information, thereby promoting innovative thinking (Gonsalves, 2026). Second, AI literacy is generated through continuous exposure to AI technology, which cultivates open acceptance and forward-looking thinking (Jiang et al., 2025). Critical and forward-looking thinking are basic characteristics of innovation. Previous studies demonstrated that high levels of digital literacy, including AI literacy, are associated with high levels of creativity and innovation among college students in relation to problem solving (Peng et al., 2026; Wang W. et al., 2026; Wang X. et al., 2026). In addition, college students with an in-depth understanding of emerging technologies are more likely to put forward new ideas and solutions in practice (Zhou et al., 2025). The results of previous studies indicate medical students who are more proficient in using digital and AI applications tend to exhibit high levels of innovation when formulating patient care plans (Wu et al., 2025).

Accordingly, drawing on the social cognitive and technology affordance theories, this study examines the promoting effect of AI literacy on innovative thinking among college students (Huang et al., 2026; Riley et al., 2016). Despite the rapid integration of AI in higher education, the mechanisms through which AI literacy influences students' innovative thinking remain insufficiently understood. Thus, the current study aims to expand this line of research by investigating how AI literacy enhances innovative thinking and by examining the impact of AI literacy on innovative thinking and related variables. Toward this end, a questionnaire survey was conducted to provide valuable information for promoting AI acceptance, enhancing social participation, and improving professional competence among college students.

## 2 Literature review and research hypotheses

### 2.1 Theoretical foundations

The proliferation of AI is profoundly transforming university students' learning methods and cognitive structures. According to Social Cognitive Theory, a dynamic interplay exists among individual behavior, cognitive states, and the environment (Riley et al., 2016). Within this framework, the use of AI tools not only alters how students acquire knowledge but also reshapes their thinking processes. As AI use increasingly transcends contextual constraints, students may develop more innovative cognitive approaches, thereby enhancing their innovative thinking.

Technology Affordance Theory emphasizes the complementary relationship between environmental properties and organism capabilities, referring to the “action possibilities” that the environment provides (Huang et al., 2026). Primarily applied in the field of human-computer interaction, it focuses on how technological features support user behavior. Within this framework, perceived ease of use and perceived usefulness exert direct effects on individuals' usage attitudes, while usage attitudes influence behavioral intentions and are also shaped by perceived usefulness. In this study, university students' perceptions of the ease of use and usefulness of AI are reflected in perceived AI interaction, which is, in turn, associated with AI attitudes.

In summary, Social Cognitive Theory explains the behavioral and cognitive foundations underlying AI use, while Technology Affordance Theory clarifies the progression from tool use to behavioral intention. Together, these theories form the analytical framework of this study and provide a strong theoretical basis for subsequent hypothesis development and model construction.

### 2.2 AI literacy and college students' innovative thinking

The current era is an intelligent one dominated by informatization and innovation, and the leap-forward development of society has increased the demand for innovative talents (Wang and Fan, 2025). Previous studies demonstrated that human production and life have undergone transformative changes with the gradual penetration of AI into society and daily life, and AI literacy has become an important component of the talent quality framework in the current era (Zhao et al., 2025). Specifically, the AI era is one of collaboration, game, and integration between human intelligence and AI (Shinkle et al., 2026). The rapid evolution and iteration of generative AI has exerted disruptive impacts on many industries (Liu and Hu, 2025). While benefiting from the advantages of AI technology, humans must also actively respond to complex problems emerging from its application. Certain studies pointed out that AI literacy can improve individuals' innovative thinking and help them solve complex problems (Hackl et al., 2026). AI literacy refers to the ability to appropriately identify, utilize, and evaluate AI-related technologies by considering ethical principles (Wang et al., 2023). A study on college students found that greater use of AI technology can improve their creativity in problem-solving (Lu et al., 2025). A major reason underlying this phenomenon may be that, through engagement with emerging technologies, college students have cultivated an open and receptive attitude toward them, thus developing innovative thinking (Liu et al., 2025a,b). Recent studies have demonstrated that high levels of digital literacy can lead to high levels of creativity in problem solving among college students (Yang et al., 2025). Despite the rapid integration of AI in higher education, the mechanisms through which AI literacy influences students' innovative thinking remain insufficiently understood. In summary, this study puts forward the following:

H1: AI literacy positively predicts college students' innovative thinking; although the bivariate association remains significant, the direct effect becomes fully mediated.

### 2.3 Mediating role of perceived AI interaction

Perceived AI interaction mainly refers to information exchange between college students and generative AI and is considered a key factor of AI systems (Wang and Chen, 2025). Users can use generative AI to interact with various sources of information, accumulating knowledge and insights and, thus, affecting usage effect (Liu et al., 2025a,b). Perceived AI interaction is widely regarded as a crucial component of effective learning, which is closely related to improvements in knowledge acquisition skills, learner participation, and cognitive development (Ren et al., 2025). In recent years, AI integration in education has introduced a new interaction mode, which is especially evident in interactions between learners and intelligent systems (Niculescu et al., 2025). The advancement of large language models has enabled AI tools to provide various functions such as personalized guidance, real-time feedback, and flexible access, which may supplement or change established learning processes (Olgen and Cucuzzella, 2025). Systems such as ChatGPT and Deep Seek have demonstrated dialogue capabilities that are very similar to human interactions in certain scenarios, which may support greater interactive learner-AI communication (Zheng, 2025). The core concept in this field is perceived AI interaction—the subjective experience of learners in the interaction process of meaningful, responsive, and regulated communication (Wang et al., 2025). With the development of AI technology, learners are increasingly becoming active participants, instead of information receivers, in communication with AI systems (Liu et al., 2025a,b).

Research on human-computer interaction skills has become an emerging research hotspot (Al-Shamaileh et al., 2026). These studies encompass AI-centered perspectives and adopt an evaluation framework that emphasizes users' understanding of AI principles and operation mechanisms (Gong et al., 2025). However, this approach overlooks human-computer interaction within general AI and underestimates the AI literacy of the majority of non-expert user groups (He, 2025). Several studies have emphasized that human-computer interaction ability is a critical behavioral dimension of AI literacy (Tully et al., 2025). In essence, interaction between humans and generative AI is similar to that between humans and other types of media: both of them aim to promote the efficient acquisition of accurate information (Wu and Guo, 2025).

Studies illustrate that the stronger the perceived AI interaction, the more positive the students' usage attitude, which positively influences innovative thinking (Sengul et al., 2025). The high interactivity of generative AI can provide students with personalized information services according tailored to prior needs and preferences (Lyu et al., 2026). Meanwhile, generative AI can continuously adjust response strategies through interaction and feedback with students. Therefore, perceived AI interaction can be an effective tool for promoting the development of innovative thinking among college students. Thus, this study presents the following:

H2: Perceived AI interaction plays a mediating role in the relationship between AI literacy and college students' innovative thinking.

### 2.4 Mediating role of AI attitudes

For college students, AI attitudes may be another core mediating factor of the effect of AI literacy on innovative thinking (Cengiz and Peker, 2025; Erciyas et al., 2024; Saklaki and Gardikiotis, 2024). As a comprehensive embodiment of cognition, operation, ethical judgment, and problem-solving ability related to AI technology, AI literacy serves as the core antecedent of AI attitudes (Bewersdorff et al., 2025). Existing studies demonstrated that technical literacy levels directly determine cognitive depth and judgment ability of technology (Katsantonis and Katsantonis, 2024). High levels of AI literacy can enhance understanding of the benefits and limitations of AI technology in an objective and comprehensive manner, thus mitigating extreme attitudes (e.g., fear of technology and blind dependence) and promoting a rational, inclusive, and proactive approach (Boztepe et al., 2025).

AI attitudes, as a comprehensive embodiment of cognitive tendency, emotional experience, and behavioral intention toward AI technologies, directly impacts the formation and development of innovative thinking (Falebita and Kok, 2025). The core of innovative thinking lies in breaking through traditional thinking patterns and actively exploring new ideas and methods, while positive attitudes toward AI can stimulate the desire for exploration and innovation willingness and encourage individuals to actively accept and use AI tools in conducting creative activities (Bayaga, 2025). Relevant studies pointed out that, within educational environments, college students with positive attitudes toward AI are more inclined to consider AI a cognitive partner; realize information integration and thinking collision through in-depth interaction; and improve levels of cognitive flexibility and innovative thinking (Lin and Chen, 2024). In contrast, negative attitudes toward AI can lead individuals to feel alienated from technology, adhere to traditional thinking modes, and hinder the development of innovative thinking. In addition, empirical studies on AI attitudes have found that positive attitudes toward technology can lend psychological support for innovation and encourage individuals to actively explore the combination point of technology and innovation (Yuanyuan et al., 2026). The formation of this positive attitude frequently depends on high levels of technical literacy (Mao et al., 2026). A number of scholars have discussed the relationship between digital literacy and innovative thinking and found that attitudes toward digital technologies is an important psychological component that bridges literacy to cognition, and the improvement of literacy levels can be more effectively applied to the development of innovation ability only through transformation in attitude (Wang W. et al., 2026; Wang X. et al., 2026). From the perspective of AI research, improving AI literacy can optimize AI attitudes, while positive technology attitude can promote the development of innovative thinking.

Previous studies have also pointed out that AI literacy exerts a significant positive effect on college students' AI attitudes (Erciyas et al., 2024; Saklaki and Gardikiotis, 2024). College students with high levels of AI literacy exhibit better attitudes toward AI and an in-depth understanding of AI at the same time. Meanwhile, AI literacy can promote the transformation of cognitive styles, which influences positive attitudes toward AI and, consequently, innovative thinking (Wang W. et al., 2026; Wang X. et al., 2026). Individuals with high levels of AI literacy tend to acquire more knowledge about AI, which, in turn, affects their attitudes toward AI (Lin and Chen, 2024). Accordingly, the current study presents the following:

H3: AI attitudes play a mediating role in the relationship between AI literacy and college students' innovative thinking.

### 2.5 Construction of the chain-based mediating model

Existing research indicates a significant association between individuals' perceived AI interaction and AI attitudes. Empirical evidence suggests that a stronger perception of AI interaction is associated with more positive usage attitudes among students, and that such attitudes, in turn, positively influence students' innovative thinking (Sengul et al., 2025).

At present, a substantial body of literature has examined the effects of perceived AI interaction on AI attitudes and related psychological outcomes (Ho et al., 2026). However, further in-depth investigation is required to clarify the mechanisms underlying these relationships. Some scholars have proposed explanatory models of perceived AI interaction, elucidating why stronger interaction perceptions can positively predict AI attitudes (Celik et al., 2026). Specifically, individuals with higher levels of perceived AI interaction tend to exhibit more positive self-cognition and greater confidence in their abilities. This enhanced self-perception encourages the setting of more ambitious goals and strengthens achievement motivation, thereby fostering a more favorable AI attitudes (Sengul et al., 2025).

From a cognitive-behavioral perspective, both perceived AI interaction and AI attitudes can be understood as reflective cognitive responses that emerge following individuals' engagement with AI-related behaviors (Sengul et al., 2025). Accordingly, it is theoretically reasonable to conceptualize these variables as mediators in the relationship between college students' AI literacy and innovative thinking in this study. By intentionally designing generative AI systems that enhance users' interaction experience, students' perceived engagement during platform use can be continuously improved. This, in turn, is expected to strengthen students' attitude toward generative AI and ultimately promote innovative thinking.

In summary, although prior studies have identified associations between AI literacy and perceived AI interaction, as well as between AI attitudes and innovative thinking, systematic examination of the internal mechanism and interactive pathways among these four variables remains limited. To address this gap, this study conceptualizes AI literacy as the independent variable, innovative thinking as the dependent variable, and perceived AI interaction and AI attitudes as chain mediating variables. Based on this framework, the following hypothesis is proposed:

H4: Perceived AI interaction and AI attitudes jointly play a chain mediating role in the relationship between AI literacy and college students' innovative thinking.

**Figure 1:**
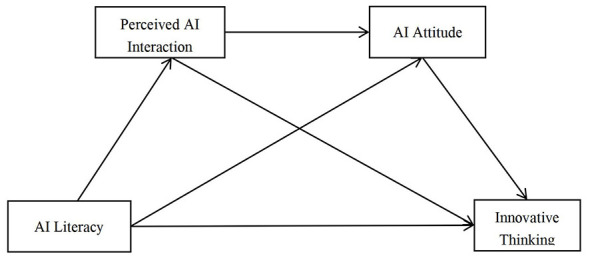
Hypothesized research model.

The hypothesized research model is presented in Figure 1.

## 3 Methods

### 3.1 Participants

An a priori sample size estimation was conducted using G^*^Power 3.1. The parameters were set as follows: effect size *f* = 0.25, significance level α = 0.05, and statistical power (1–β) = 0.95, resulting in a minimum required sample size of 210 participants. This study employed a stratified random sampling approach, recruiting students from four universities in Shandong, Ningxia, Hainan, and Jiangxi. A total of 1,000 university students were invited to complete an online questionnaire to ensure adequate statistical power. Two of the participating institutions were private universities. To enhance sample diversity and representativeness, participants were stratified by academic year and major within each institution. A simple random sampling procedure was then applied within each stratum to select respondents, thereby improving the overall representativeness of the sample. This study was approved by the Ethics Committee of Shandong Sport University (Approval No.: 2025085). Prior to data collection, the researchers obtained informed consent from the participants. They were assured that participation was in the survey was anonymous, and the data would be used only for scientific research purposes and treated with strict confidentiality. This study followed the Declaration of Helsinki.

The participants were screened using clear inclusion and exclusion criteria to ensure the accuracy and generalizability of the study. The inclusion criteria were college students who were enrolled full time, without physical disabilities, can participate in relevant activities, and did not previously participate in similar studies. The exclusion criteria included failing to meet the inclusion criteria, unreliable responses (e.g., consistent answers across all items or a response time of less than 1 min), and incomplete questionnaires. Wenjuanxing, a Chinese online survey platform similar to Amazon's Mechanical Turk, was selected for questionnaire dissemination and collection. Prior to the survey, the participants were fully informed regarding anonymity, confidentiality rules, and research objective. After screening, a total of 1,000 questionnaires were distributed, 34 questionnaires considered invalid were excluded, and 966 valid questionnaires were recovered, with an effective response rate of 96.6%. Among the valid samples, 437 were men (45.2%), while 529 were women (54.8%). In terms of education, 336 (34.8%), 373 (38.6%), 179 (18.5%), and 78 (8.1%) were freshmen, sophomores, juniors, and seniors, respectively.

### 3.2 Instruments

#### 3.2.1 Artificial intelligence literacy scale

This study used the Artificial Intelligence Literacy Scale to measure levels of AI literacy (Wang et al., 2023). The scale comprises four dimensions—AI awareness, AI usage, AI evaluation, and AI ethics—each containing three items. In total, the scale includes 12 items rated on a five-point Likert scale ranging from 1 (“strongly disagree”) to 5 (“strongly agree”). Higher average scores indicate higher levels of AI literacy among university students. The scale demonstrated satisfactory model fit indices (CFI = 0.99, TLI = 0.99, GFI = 0.98, RMSEA = 0.01, SRMR = 0.03). Cronbach's α coefficient was 0.866, indicating acceptable internal consistency reliability.

#### 3.2.2 Innovative thinking scale

To measure levels of innovative thinking, this study used the Innovative Thinking Scale (Morad et al., 2021). The scale contains 19 items across four dimensions: observation, questioning, creative networking, and experimentation. Items were rated on a five-point Likert scale, with higher average scores indicating higher levels of innovative thinking. Cronbach's α coefficient was 0.733, and model fit indices were satisfactory (NFI = 0.92, TLI = 0.93, CFI = 0.94, RMSEA = 0.07, SRMR = 0.04).

#### 3.2.3 Perceived AI interaction scale

The study employed the Perceived AI Interaction Scale to assess levels of perceived AI interaction (Wang et al., 2025). The scale consists of 17 items covering responsiveness, controllability, participation, and personalization. Items were rated using a seven-point Likert scale. The scale demonstrated satisfactory model fit indices (χ^2^/df = 2.148, RMSEA = 0.061, SRMR = 0.062, TLI = 0.926, CFI = 0.938). Cronbach's α coefficient was 0.977, indicating excellent internal consistency.

#### 3.2.4 AI attitude scale

The AI Attitude Scale was used to measure AI attitudes (Talik et al., 2025). This scale has demonstrated good reliability and validity in samples of college students from Poland, the United Kingdom, and the United States. Items were rated on a five-point Likert scale, with higher average scores indicating more positive attitudes toward AI. Cronbach's α coefficient was 0.946, and the model fit indices were satisfactory (χ^2^/df = 2.49, CFI = 0.999, TLI = 0.998, RMSEA = 0.0285).

### 3.3 Data processing and analysis

SPSS 26.0 was used for statistical analysis. First, given the use of the mixed Likert scale method bias research design and measurement, the common method bias test was conducted to control for systematic measurement error (Podsakoff et al., 2003). Second, analysis of variance (ANOVA) was performed to examine differences in AI literacy, innovative thinking, perceived AI interaction, and AI attitudes among college students with various demographic characteristics. Third, correlation analysis was conducted to explore bivariate associations among the four core variables. Finally, PROCESS macro (Hayes) was used to test the proposed chain mediation model, and the bootstrap method with 5,000 iterations was employed to determine the significance of different mediation paths.

## 4 Results

### 4.1 Common method bias test

To ensure that common method bias did not threaten the validity of the findings, this study employed both procedural remedies and statistical tests. As a procedural control, anonymous participation was implemented to reduce respondents' evaluation apprehension and, consequently, minimize the risk of common method bias (Podsakoff et al., 2003).

Harman's single-factor test was adopted to test common method bias for all items of the measurement tools. The results demonstrated that 7 factors obtained eigenvalues greater than 1, and the average variance extracted of the first factor was 30.552%, which was lower than the threshold (40%).

In addition, this study employed the unmeasured latent methods construct technique to test for common method bias. The key model fit indices for the comparison were: ΔGFI = 0.005, ΔIFI = 0.002, ΔNFI = 0.008, and ΔRMSEA = 0.003. All changes in fit indices were below 0.03, indicating that the model did not improve substantially after adding the common method factor. In conclusion, this study does not suffer from serious common method bias (Podsakoff et al., 2003).

To further assess common method bias, variance inflation factors (VIFs) for all latent variables were calculated. The VIF values were 1.251, 1.957, and 2.082, which were all below the recommended cutoff value of 3 (Kock, 2015), indicating the absence of substantial collinearity-based common method bias.

### 4.2 Difference analysis on demographic variables

The study conducted *t*-test to compare scores for AI literacy, innovative thinking, perceived AI interaction, and AI attitudes among college students across two groups with different demographic characteristics. Additionally, ANOVA was performed to compare differences among multiple groups. The results demonstrated a statistically significant gender difference in AI attitudes, with female students exhibiting higher scores. In addition, a significant difference in year level was observed for perceived AI interaction, with the overall trend: the higher the grade, the higher the level of perceived AI interaction (Table 1).

**Table 1:** Differences by gender and grade.

Variable	Groups	Statistics	AI literacy	Innovative thinking	Perceived AI interaction	AI attitudes
Gender	MMen (437)		3.28 ± 0.69	4.01 ± 0.38	3.79 ± 1.77	2.99 ± 1.37
	FWomen (529)		3.31 ± 0.72	3.97 ± 0.34	3.62 ± 2.04	3.23 ± 1.38
		*t*	−0.625	1.518	1.366	−2.664
		*p*	0.532	0.129	0.172	0.008
Year level	1		3.33 ± 0.74	4.00 ± 0.37	3.54 ± 1.94	3.07 ± 1.40
	2		3.29 ± 0.70	3.98 ± 0.35	3.77 ± 1.87	3.18 ± 1.37
	3		3.26 ± 0.69	3.98 ± 0.34	3.59 ± 2.05	3.05 ± 1.41
	4		3.29 ± 0.62	3.98 ± 0.33	4.24 ± 1.68	3.19 ± 1.34
		*F*	0.504	0.261	3.241	0.591
		*p*	0.679	0.854	0.021	0.621

### 4.3 Descriptive statistical analysis of variables

Pearson product-moment correlation analysis was conducted to examine the relationships among AI literacy, innovative thinking, perceived AI interaction, and AI attitudes. The results are presented in Table 1. AI literacy showed a significant positive correlation with innovative thinking (*r* = 0.139, *p* < 0.001), as well as significant positive correlations with perceived AI interaction (*r* = 0.366, *p* < 0.001) and AI attitudes (*r* = 0.436, *p* < 0.001). Innovative thinking was also significantly positively correlated with perceived AI interaction (*r* = 0.222, *p* < 0.001) and AI attitudes (*r* = 0.223, *p* < 0.001). Finally, perceived AI interaction exhibited a significant positive correlation with AI attitudes (*r* = 0.683, *p* < 0.001; see Table 2).

**Table 2:** Descriptive statistics of variables and correlation analysis between variables (*r*).

Variable	*M*	*SD*	1	2	3	4
1. AI literacy	3.30	0.71	1			
2. Innovative thinking	3.99	0.35	0.139^***^	1		
3. Perceived AI interaction	3.69	1.92	0.366^***^	0.222^***^	1	
4. AI attitudes	3.12	1.38	0.436^***^	0.223^***^	0.683^***^	1

^***^p < 0.001, same below.

### 4.4 Chain mediating effects of perceived AI interaction and AI attitudes in the relationship between AI literacy and innovative thinking

Given the significant correlations among AI literacy, innovative thinking, perceived AI interaction, and AI attitudes, as well as the absence of multicollinearity, the data met the assumptions required for mediation analysis. All variables were standardized prior to analysis. The PROCESS macro in SPSS was used to test the hypothesized chain mediation model, with gender and grade included as control variables. A total of 5,000 bootstrap samples were used to estimate the significance of indirect effects. The model was specified as follows: Model 6; X = AI literacy, M1 = perceived AI interaction, M2 = AI attitudes, and Y = innovative thinking. Table 2 presents the results. Prior to the addition of the mediating variables, the direct path from AI literacy to innovative thinking was significant (β = 0.140, *t* = 4.381, *p* < 0.001), which indicates that AI literacy positively predicts innovative thinking. Thus, H1 was supported.

**Figure 2:**
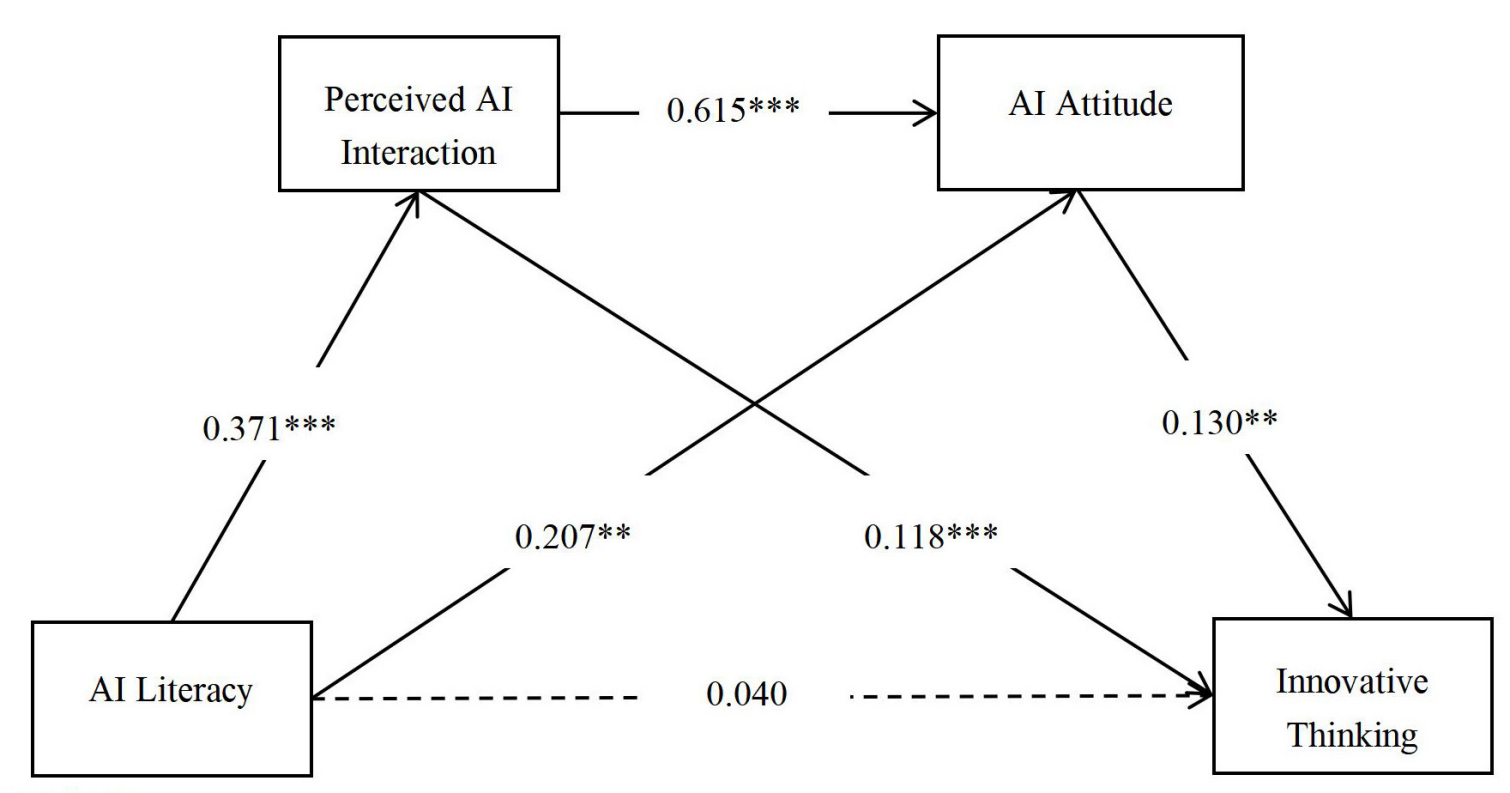
Chain mediation model.

The study also conducted a chain mediation analysis with AI literacy as the predictive variable, innovative thinking as the outcome variable, and perceived AI interaction and AI attitudes as the mediating variables. Table 3 and Figure 2 presents the results. All direct paths in the model reached a significant level (*p* < 0.001); in the mediating path with perceived AI interaction as the mediating variable, AI literacy significantly positively predicted perceived AI interaction (β = 0.371, *t* = 12.414, 95% CI: [0.850, 1.169]), which, in turn, positively predicted innovative thinking (β = 0.118, *t* = 2.696, 95% CI: [0.006, 0.038]); in the mediating path with AI attitudes as the mediating variable, AI literacy significantly positively predicted AI attitudes (β = 0.207, *t* = 8.583, 95% CI: [0.313, 0.498]), which, in turn, also positively predicted innovative thinking (β = 0.130, *t* = 2.891, 95% CI: [0.011, 0.056]). Lastly, in the chain mediating path from perceived AI interaction to AI attitudes perceived AI interaction positively predicted AI attitudes (β = 0.615, *t* = 25.449, 95% CI: [0.408, 0.476]).

**Table 3:** Regression analysis of variables.

Variables	Innovative thinking		Perceived AI interaction		AI attitudes		Innovative thinking	
	β	***t***	β	***t***	β	***t***	β	***t***
Gender	−0.050	−1.551	−0.063	−2.086^*^	0.113	5.012^***^	−0.052	−1.633
Grade	−0.014	−0.433	0.089	2.964	−0.038	−1.693	−0.027	−0.839
AI literacy	0.140	4.381^***^	0.371	12.414^***^	0.207	8.583^***^	0.040	1.132
Perceived AI interaction					0.615	25.449^***^	0.118	2.696^**^
AI attitudes							0.130	2.891^**^
*R*	0.149		0.380		0.721		0.253	
*R^2^*	0.022		0.145		0.520		0.064	
*F*	7.298		54.202		259.959		13.106	

^*^p < 0.05, ^**^p < 0.01, ^***^p < 0.001, same below.

The bias-corrected nonparametric percentile bootstrap method (with 5,000 resampling iterations) was used to test the significance level of the multiple mediating effects of the model. If the 95% CI of a mediating effect path does not include 0, then the mediating effect of the path is significant, and the mediating path consists of the indirect effects generated by each subpath (Table 4). Notably, the total effect in Table 4 (0.070) differs from the zero-order β in Table 3 (0.140), suggesting the effect after full mediation by the chained variable. First, perceived AI interaction mediated the relationship between AI literacy and innovative thinking (β = 0.023, 95% CI = [0.008, 0.040]), accounting for 32.86% of the total effect. Second, AI attitudes mediated the relationship between AI literacy and innovative thinking (β = 0.013, 95% CI = [0.003, 0.024]), accounting for 18.57% of the total effect. Third, perceived AI interaction and AI attitudes jointly formed a chain mediation pathway between AI literacy and innovative thinking (β = 0.013, 95% CI = [0.003, 0.025]), accounting for 18.57% of the total effect. The results support H2–H4.

**Table 4:** Bootstrap analysis of the significance test of mediating effects.

Impact pathways	Effect	Boot SE	Boot LLCI	Boot ULCI	Effect size ratio
Total effect	0.070	0.016	0.039	0.101	
Direct effect	0.021	0.018	−0.014	0.055	
Total indirect effect	0.049	0.008	0.033	0.066	70%
Ind1(AL → PAI → IT)	0.023	0.008	0.008	0.040	32.86%
Ind2(AL → AA → IT)	0.013	0.005	0.003	0.024	18.57%
Ind3(AL → PAI → AA → IT)	0.013	0.005	0.003	0.024	18.57%
C1(Ind1-Ind2)	0.010	0.012	−0.013	0.035	
C2(Ind1-Ind3)	0.010	0.012	−0.013	0.034	
C3(Ind2-Ind3)	−0.0004	0.002	−0.005	0.004	

AL, AI Literacy; PAI, Perceived AI Interaction; AA, AI Attitudes; IT, Innovative Thinking.

## 5 Discussion

### 5.1 Analysis of the relationship between AI literacy and innovative thinking

The results of correlation analysis and regression model demonstrate that AI literacy is closely correlated with and can effectively improve innovative thinking, which is consistent with finding from previous studies. Moreover, the results supported H1 (Lu et al., 2025; Yang et al., 2025). Therefore, the concept of AI literacy, including technical cognition, tool application, information integration, critical thinking, and problem-solving skills, is highly consistent with the core dimensions of innovative thinking such as divergence, flexibility and originality. College students expand their cognitive boundaries, optimize thinking processes, and overcome the limitations of traditional thinking through AI technologies, thus promoting the generation of innovative ideas and improvement in creative problem-solving skills. AI literacy has become a critical foundation of the development of college students' innovation ability in the digital age. In the process of cultivating innovative talents, the need emerges for colleges and universities to incorporate AI literacy into curricula; improve students' comprehension and application levels of AI technologies through teaching, practical training, and interdisciplinary integration; and foster the development of innovative thinking with technical literacy, to provide effective paths for improvement in innovation ability.

### 5.2 Analysis of the mediating role of perceived AI interaction

Consistent with H2, the study finds that perceived AI interaction plays a mediating role in the relationship between AI literacy and innovative thinking (Tully et al., 2025). Specifically, alongside subjective experiences and perceptions formed in the process of interaction with AI technology, perceived AI interaction is a key factor that links AI literacy and innovative thinking. College students with high levels of AI literacy can interact with AI tools in a more skillful and effective manner. In a process of active exploration, trial application, and feedback adjustment, they gradually form positive interactive experiences, which can stimulate their desire to explore and seek knowledge, deviate from traditional cognition patterns, and view problems from multiple perspectives.

Specifically, high levels of AI literacy enable college students to accurately comprehend the functions and application scenarios of AI tools, thus making it easier to match their needs with tools in the interaction process. Consequently, negative experiences caused by operational obstacles are reduced, and their confidence and initiative in using technology to solve problems are enhanced. This positive perception of AI interaction could encourage college students to conduct exploratory learning and thinking collision using AI tools, gradually cultivate divergent and original thinking, and, finally, achieve improvement in innovative thinking. The discovery of this mediating effect enriches the theoretical path of AI literacy in relation to innovative thinking, which indicates that the effect of AI literacy on innovative thinking is not a single direct effect but realized through the mediating role of interactive experience. The results also provide a targeted framework that colleges and universities can use for innovative talent cultivation: they should focus on providing students with guidance in actively participating in AI interaction practices and optimizing interaction experiences, while cultivating their AI literacy, to maximize the potential of the promoting effect of AI literacy on innovative thinking.

### 5.3 Analysis of the mediating role of AI attitudes

Consistent with H3, AI attitudes play a mediating role in the relationship between AI literacy and innovative thinking, which is consistent with the literature (Cengiz and Peker, 2025). As a comprehensive embodiment of cognitive tendency, emotional experience, and behavioral intention toward AI technology, AI attitudes serve a crucial psychological medium for the transformation of AI literacy into innovative thinking. College students with high levels of AI literacy can recognize the benefits and limitations of AI technology objectively and comprehensively and are, thus, less likely to possess extreme attitudes such as technophobia or blind reliance, which leads to the formation of a rational, inclusive, and proactive AI attitudes. This positive attitude could encourage college students to accept and apply AI technology and actively explore combination points of technology and innovation in learning and practice, thereby stimulating innovative thinking. These positive AI attitudes can effectively stimulate motivation of students toward innovation and encourage them to view AI technology as an innovation tool instead of passively accept technical services. In learning and practice, college students with positive attitudes exhibit more willingness to explore and combine AI with professional learning and innovation practice, constantly examine new ideas and methods in technology application, and improve the originality and flexibility of their thinking. This result further enriches the theoretical cognition of innovative talent cultivation in the digital age and elucidates the key role of AI attitudes in the transformation from AI literacy to innovative thinking. At the practical level, when conducting AI literacy education, colleges and universities should not only pay attention to the teaching of technical skills but also strengthen the guidance of college students' AI attitudes, enable students to establish rational and positive attitudes toward AI using various methods, overcome psychological barriers in the process of literacy transformation, and promote AI literacy as a strong driving force for the improvement of innovative thinking.

### 5.4 Chain mediating effect of perceived AI interaction and AI attitudes

As shown in H4, perceived AI interaction and AI attitudes play a chain mediating effect on the relationship between AI literacy and innovative thinking (Sengul et al., 2025). Validating this chain mediating effect further improves the model of the relationship between AI literacy and innovative thinking, which indicates that the effect of AI literacy on innovative thinking should not only be transmitted at the behavioral level of interactive experiences but should also be transformed at the psychological level of attitude cognition. This aspect provides detailed practical guidance for the cultivation of innovative talents in colleges and universities. Specifically, on the basis of cultivating AI literacy, colleges and universities should provide students with guidance not only in actively participating in AI interaction practice and optimize interaction experiences but also in establishing positive and rational attitudes toward AI, thereby fostering the development of innovative thinking.

From a theoretical perspective, these findings support the integrative logic of Social Cognitive Theory and Technology Affordance Theory: as AI tools transform the ways in which students acquire knowledge, more innovative cognitive processes emerge, and perceived AI interaction and AI attitudes influence individual behavioral intentions. From a practical perspective, this study suggests that educational administrators should emphasize human-AI collaborative thinking frameworks in AI-enhanced instruction. Universities should design curricula oriented toward problem-solving and innovative learning, while encouraging students to engage in critical scrutiny and self-correction when using AI, thereby enabling the effective and meaningful use of AI technologies to support cognitive development.

Perceived AI interaction and AI attitudes play a significant chain mediating role in the relationship between AI literacy and innovative thinking: AI literacy influences innovative thinking through the chain path of perceived AI interaction and AI attitudes. This result further improves the internal action mechanism of the relationship between AI literacy and innovative thinking and reveals the influence path between them. The existence of this chain mediating effect indicates that the effect of AI literacy on innovative thinking is not a single link transmission but realized through the progressive transformation of interactive experiences and cognitive attitudes. The discovery of this chain mediating mechanism could serve as reference for colleges and universities in cultivating innovative thinking. Guiding college students in actively participating in AI interaction practice and fostering positive attitudes toward AI are necessary steps to maximize the impact of AI literacy on innovative thinking.

## 6 Conclusion

This study, based on a sample of 966 university students, systematically examined the mechanisms through which AI literacy, perceived AI interaction, and AI attitudes influence students' innovative thinking. It further analyzed the optimal pathways for enhancing innovative thinking. The results indicate that AI literacy can significantly positively predict the innovative thinking. Moreover, AI literacy influences innovative thinking not only through the independent mediating roles of perceived AI interaction and AI attitudes but also through their sequential chain mediation, which fully mediates the relationship between AI literacy and innovative thinking. These findings suggest that improving AI literacy is an effective strategy for enhancing university students' innovative thinking; however, strengthening perceived AI interaction and AI attitudes may further amplify this effect.

From a theoretical perspective, this study integrates the social cognitive and technology affordance theories to construct a framework that links AI literacy, perceived AI interaction, AI attitudes, and innovative thinking. The findings underscore that the role of AI in education is not unidirectional; rather, its impact depends on the interaction between external environmental perceptions and individual cognitive states. From a practical perspective, educators should guide students to use AI tools purposefully while fostering positive AI interaction perception and AI attitudes. Universities should also optimize academic evaluation systems and cultivate supportive learning environments to enhance students' creativity and sustain their innovative capacity.

## 7 Limitations

This study employed a cross-sectional research design, which can reveal the status quo at a specific point in time but struggles to capture the dynamic relationships between variables over time. In particular, it imposes limitations on inferring long-term effects and causal relationships. Although our sample included college students of different genders and grade levels, this provided favorable conditions for obtaining diverse perspectives. However, we must recognize that this may introduce selection bias due to the use of stratified random sampling. This limitation somewhat reduces the generalizability of the findings, which may not represent the entire college student population. This study primarily relies on self-reported data collection methods, which are susceptible to subjective factors and may introduce certain cognitive biases or recall biases, thereby affecting the objectivity and accuracy of the data.

The final model demonstrated limited explanatory power, primarily because innovative thinking is a complex outcome shaped by multidimensional and multilevel factors. The sample of this study comprised 966 college students. The results revealed a significant positive correlation between AI literacy and innovative thinking (*r* = 0.139, *p* < 0.001), albeit with a small effect size. Future research should note that larger samples are more likely to detect weak statistical correlations, enabling even trivial associations to reach highly significant levels; however, such statistical significance does not necessarily imply a substantial practical effect magnitude. The relatively narrow range of variables included in this study constrained the model's overall explanatory capacity. While this limitation is acknowledged, it does not diminish the study's research value. The primary contribution lies in clarifying the pathways and boundary conditions among the variables, confirming the significant chain mediating effects, and providing incremental theoretical insights into the mechanisms underlying the development of innovative thinking.

## Data Availability

The original contributions presented in the study are included in the article/supplementary material, further inquiries can be directed to the corresponding author/s.
